# Identifying targets of the Sox domain protein Dichaete in the *Drosophila* CNS via targeted expression of dominant negative proteins

**DOI:** 10.1186/1471-213X-13-1

**Published:** 2013-01-05

**Authors:** Shih Pei Shen, Jelena Aleksic, Steven Russell

**Affiliations:** 1 Department of Genetics, University of Cambridge, Cambridge, UK; 2 Cambridge Systems Biology Centre, University of Cambridge, Cambridge, UK; 3 Present address: Wellcome Trust Sanger Institute, Wellcome Trust Genome Campus, Hinxton, UK

**Keywords:** Sox, Dichaete, Drosophila, CNS, Dominant-negative, Genomics

## Abstract

**Background:**

Group B Sox domain transcription factors play important roles in metazoan central nervous system development. They are, however, difficult to study as mutations often have pleiotropic effects and other Sox family members can mask phenotypes due to functional compensation. In *Drosophila melanogaster*, the Sox gene *Dichaete* is dynamically expressed in the embryonic CNS, where it is known to have functional roles in neuroblasts and the ventral midline. In this study, we use inducible dominant negative proteins in combination with ChIP, immunohistochemistry and genome-wide expression profiling to further dissect the role of *Dichaete* in these two tissues.

**Results:**

We generated two dominant negative *Dichaete* constructs, one lacking a DNA binding domain and the other fused to the Engrailed transcriptional repressor domain. We expressed these tissue-specifically in the midline and in neuroblasts using the UAS/GAL4 system, validating their use at the phenotypic level and with known target genes. Using ChIP and immunohistochemistry, we identified two new likely direct Dichaete target genes, *commisureless* in the midline and *asense* in the neuroectoderm. We performed genome-wide expression profiling in stage 8–9 embryos, identifying almost a thousand potential tissue-specific Dichaete targets, with half of these genes showing evidence of Dichaete binding *in vivo*. These include a number of genes with known roles in CNS development, including several components of the Notch, Wnt and EGFR signalling pathways.

**Conclusions:**

As well as identifying *commisureless* as a target, our data indicate that Dichaete helps establish its expression during early midline development but has less effect on its established later expression, highlighting Dichaete action on tissue specific enhancers. An analysis of the broader range of candidate Dichaete targets indicates that Dichaete plays diverse roles in CNS development, with the 500 or so Dichaete-bound putative targets including a number of transcription factors, signalling pathway components and terminal differentiation genes. In the early neurectoderm we implicate Dichaete in the lateral inhibition pathway and show that Dichaete acts to repress the proneural gene *asense*. Our analysis also reveals that dominant negatives cause off-target effects, highlighting the need to use other experimental data for validating findings from dominant negative studies.

## Background

During the process of development, precise changes in gene expression are critical for correctly timed differentiation and body plan development. Tissue-specific gene expression is often highly conserved across distantly related species and changes in gene expression are thought to contribute to speciation and the evolution of phenotypic diversity [1]. However, the search for direct targets of the transcription factors regulating developmental processes and the identification of regulatory networks remains challenging. In particular, it is difficult to differentiate between downstream effects, functional compensation by related genes and genuine direct targets. In the context of conserved families of transcriptional regulators, research in model organisms utilizing a number of different genetic tools can be helpful in elucidating the precise regulatory mechanisms at play in specific tissues with the hope that such insights are generalisable to a broader range of species.

Group B Sox domain factors represent such a family of conserved transcription factors with roles in a number of important developmental processes, including the regulation of stem cell pluripotency [2], eye development [3], and *Drosophila* segmentation [4,5] among others. In the context of the central nervous system, early expression of group B *Sox* genes in the neuroectoderm is detected in all metazoans examined to date, and in at least some cases it is known that this expression is functionally important [6-8]. However, a degree of functional redundancy between different *Sox* genes expressed in the same tissues makes this a difficult gene family to study and means that comparatively few direct regulatory targets are known despite the importance of this class of transcription factor [9-11]. In *Drosophila* there are four group B genes: *SoxNeuro* (*SoxN*), *Dichaete, Sox21a* and *Sox21b*, the last three organised in a genomic cluster that is conserved in insects [12-14]. *SoxN* and *Dichaete* encode group B1 proteins corresponding to mammalian Sox1 and Sox2, respectively [7]. We have previously shown that the mouse *Sox2* gene is able to effectively rescue *Dichaete* mutant phenotypes, highlighting the considerable functional conservation exhibited by Sox proteins [15].

*Dichaete* has an early role in embryonic segmentation, where it acts to regulate the expression of primary pair rule genes, and is then active in the anlage of the central nervous system [4,5]. In the early stages of *Drosophila* CNS specification, Dichaete and SoxN are required for the correct expression of the proneural gene *achaete* and in this role they cooperate with the DV-patterning homeodomain proteins Intermediate Neuroblasts Defective (Ind) and Ventral Neuroblasts Defective (Vnd) [16,17]. After gastrulation, *Dichaete* is dynamically expressed in many segregated neuroblasts and their progeny [15,18]. In the developing CNS, *Dichaete* has a defined phenotype in the ventral midline where it is a uniquely expressed *Sox* gene early in embryogenesis. It is required for the correct specification and development of midline glial cells, directly regulating the *slit* gene via interaction with the POU domain protein Ventral Veins Lacking (Vvl) and the PAS domain protein encoded by *single minded*[15,19]. Interestingly, a Sox-POU interaction is also a critical part of early mouse development, where Sox2 partners with Oct4 [20,21]. While the precise role Dichaete plays during the later stages of embryonic CNS development has not yet been clarified, a recent study suggests a role in maintaining the stem cell-like neuroblasts in a self-renewing state, with downregulation of Dichaete leading to exit from the cell cycle and premature differentiation, again showing similarity to the function of *Sox2* in mammals [22].

*Dichaete* and *SoxN* are both known to be active in the early neuroectoderm and, similarly to mammalian group B genes, display a degree of functional redundancy or compensation. Mutations in either of these genes exhibit relatively weak phenotypes in cells where they are co-expressed but double mutants show severe neural hypoplasia, suggesting that at least one group B gene is necessary for the correct specification or differentiation of early neural progenitors [9,10]. However, while it is clear the fly group B Sox proteins can functionally compensate in regions of the neuroectoderm, a careful analysis of the phenotypes suggests that each protein has a unique role, even in cells that they are co-expressed in. This is most apparent in the intermediate column of the neuroectoderm where Dichaete acts to repress the expression of the proneural gene *achaete* (*ac*) whereas SoxN appears to have activating functions [10,16].

To date, the number of characterised direct targets of *Dichaete* in the developing CNS is comparatively small, including the proneural gene *ac* in the neuroectoderm and *slit* in the midline [10,15,16,19]. The early embryo genome-wide binding profiles published by the Berkeley Drosophila Transcription Network Project (BDTNP) and the modENCODE consortium identify Dichaete binding at thousands of genomic locations, suggesting a considerable number of direct target genes [23,24]. However, since the binding profiles were obtained using whole embryos, they do not give insight into tissue-specific aspects of *Dichaete* function. Similarly, due to functional compensation and pleiotropy it is also difficult to tease apart tissue-specific aspects of Dichaete function from the genomic analysis of loss-of-function mutants.

In this study, we probed tissue-specific *Dichaete* function *in vivo,* using two newly developed dominant negative *Dichaete* alleles. We focused on specific neural tissues in the early embryo: the neuroectoderm and the ventral midline. We first characterised the constructs using phenotypic analysis to determine whether their overall effect matches that of *Dichaete* mutants. We then used a mixture of phenotypic and binding analysis to characterise two new putative Dichaete direct target genes, *commisureless* (comm) in the midline and *asense* (*ase*) in the neuroectoderm. We showed that Dichaete directly binds to regulatory elements associated with these genes. Finally, we expressed the dominant negatives in specific neural tissues and performed whole transcriptome expression profiling, comparing these data with results from existing genome-wide binding studies to identify a set of potential Dichaete targets. Using these data, we also assess the appropriateness of dominant negatives as a tool for unravelling tissue-specific transcription factor function.

## Results

### Evaluation of dominant negative constructs

Since *Dichaete* is widely and dynamically expressed during *Drosophila* development and null mutations have pleiotropic effects [15,18], its function in specific tissues is difficult to study in detail. To facilitate tissue-specific analysis of Dichaete action, we generated dominant negative (DN) Dichaete proteins and expressed them in specific tissues via the GAL4-UAS system [25]. We produced three types of construct: two dominant negative proteins, either lacking the Dichaete HMG DNA-binding domain (ΔHMG) or a fusion between the full length Dichaete and the strong repressor domain from the Engrailed protein (EnRep), we also produced a full length wild-type Dichaete control. Both types of dominant negative protein have been previously reported in work with vertebrate group B Sox proteins: the EnRep in Xenopus [26], Chick [27] and zebrafish [28], and the ΔHMG in Xenopus [26]. EnRep fusions are reported to turn transcription factors into strong repressors, effectively shutting down the expression of target genes [29,30]. The way that ΔHMG proteins act is less clear but it is likely they function by sequestering essential co-factors. Thus our expectations when using the DN constructs was that the EnRep fusion would reveal sets of genes where Dichaete function is required for activation but would not detect genes where Dichaete normally acts as a repressor, whereas the ΔHMG construct should uncover genes repressed or activated by Dichaete *via* interactions with a cofactor. Since we have previously shown that Sox2 effectively rescues *Dichaete* mutant phenotypes, we also generated a similar set of dominant negative constructs using mouse Sox2 (mSox2) to test the generality of our approach.

**Figure 1:**
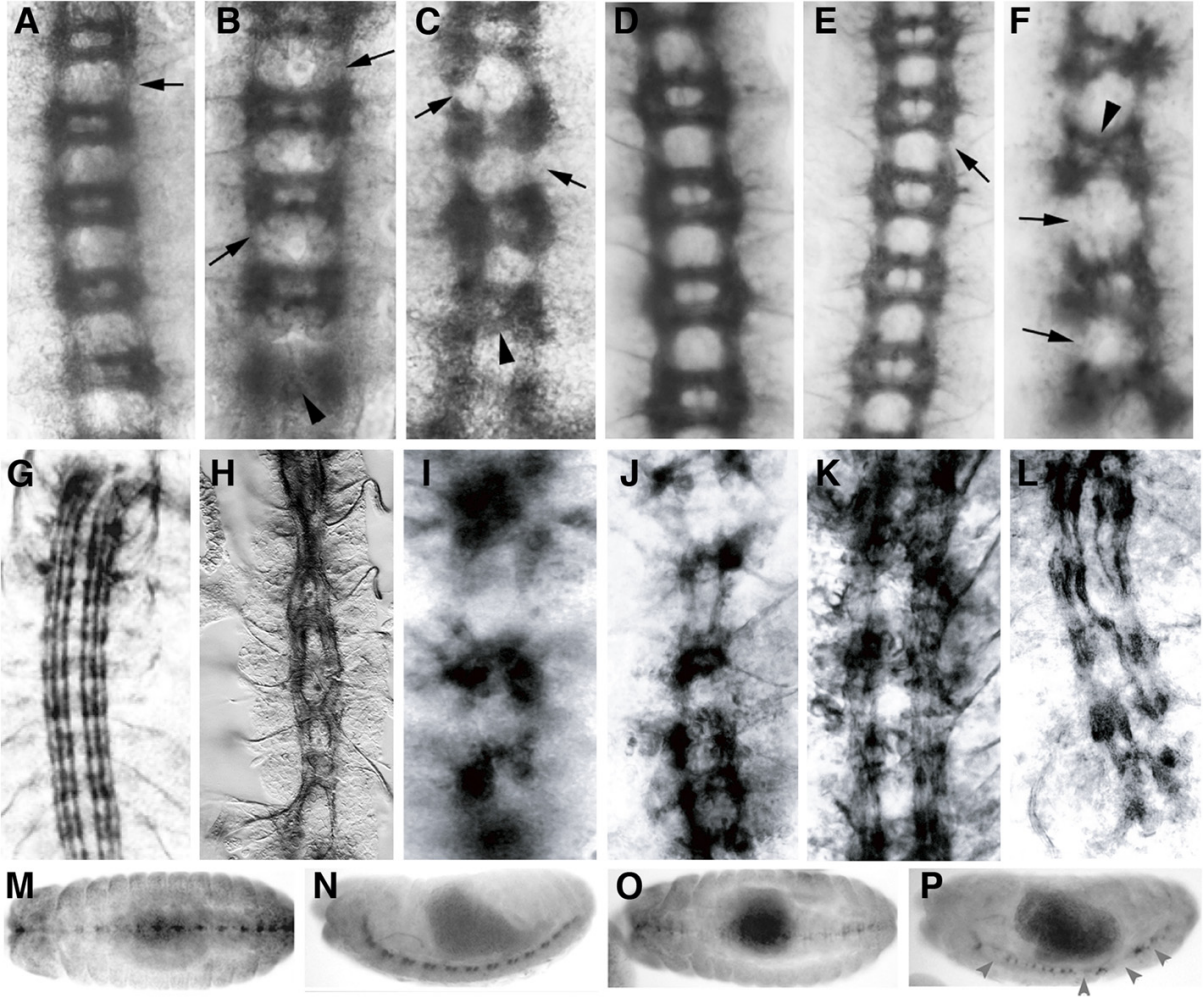
**Dominant negative proteins mimic Dichaete phenotypes. A-F**) BP102 staining reveals the ventral nerve cord of stage 16 embryos. All ventral views with anterior to the top. **A**) *sim-GAL4*;*UAS-D*; **B**) *sim-GAL4*;*UAS-D*^*ΔHMG*^; **C**) *sim-GAL4*;*UAS-D*^*EnRep*^; **D**) *pros-GAL4*;*UAS-D*; **E**) *pros-GAL4*;*UAS-D*^*ΔHMG*^; **F**) *pros-GAL4*;*UAS-D*^*EnRep*^. Arrows indicate breaks in the longitudinal connectives, arrowheads indicate collapse of commissures. **G-L**) FasII staining revealing the major longitudinal fascicles **G**) Wild type; **H**) *D*^*r72*^*/Df(3L)fz-GS1a*; **I**) *sim-GAL4*;*UAS-D*^*ΔHMG*^; **J**) *sim-GAL4*;*UAS-D*^*EnRep*^; **K**) *sim-GAL4*;*UAS-mSox2*^*ΔHMG*^; **L**) *sim-GAL4*;*UAS-mSox2*^*EnRep*^. **M-P**) Anti-Slit staining to reveal the midline glia. **M and N**) Wild type at stage 13 (M) and 15 (N). **O and P**) *sim-GAL4*;*UAS-D*^*ΔHMG*^ at stage 13 (O) and 15 (P). M and O are ventral views with anterior to the left, N and P are lateral views with anterior to the left. Grey arrowheads indicate loss of Slit expression.

To assess the effectiveness of the DN constructs we expressed each of them in the midline of wild type flies using a *sim*-GAL4 driver and in neuroblasts using a *pros-*GAL4 driver. In the case of *sim-GAL4* we used a driver line heterozygous for *sim-*GAL4 on the 2nd and homozygous for *sim-*GAL4 on the 3rd chromosome crossed to homozygous UAS lines. For *pros-*GAL4, a 2nd chromosome homozygous driver line was crossed with homozygous UAS lines. In both cases, all progeny express the transgene. We have previously shown that expressing wild type Dichaete or mSox2 with a midline driver rescues *Dichaete* null mutant phenotypes [15]: both Dichaete ΔHMG and EnRep constructs produced *Dichaete-*like phenotypes in the CNS when expressed with *sim-GAL4*. BP102 staining of DN-Dichaete expressing embryos showed collapsed commissures, reduced separation between the longitudinal tracts, gaps in longitudinal connectives, misrouting and inappropriate midline crossing by longitudinal axons (Figure 1A-F). Similar phenotypes were observed with the mSox2 constructs (not shown) and in all cases 50-70% of embryos (n > 500) exhibited some degree of CNS disruption. In agreement with our previous observations [15], expression of the wild type Sox proteins under the same conditions produced very mild phenotypes (Figure 1A and D). Focusing on the longitudinal connectives as revealed by FasII staining, we observed strong CNS phenotypes with both the fly and mouse DN constructs (Figure 1G-L).

**Figure 2:**
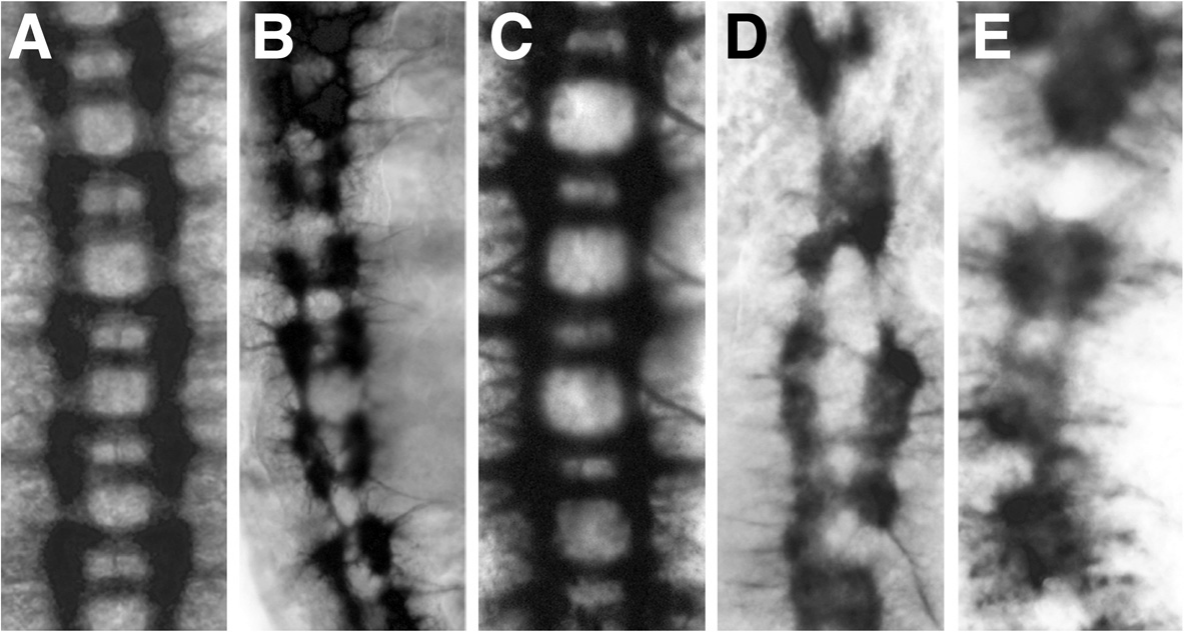
**Dominant negative expression in a *Dichaete *mutant background. A-E**) BP102 staining, all embryos oriented with anterior to the top. **A**) Wild type; **B**) *D*^*r72*^*/Df(3L)GS1a; ***C**) *D*^*r72*^*/Df(3L)GS1a, simGAL4/UAS-mSox2*; **D**) *D*^*r72*^*/Df(3L)GS1a; simGAL4/UAS-mSox2*^*ΔHMG*^; **E**) *D*^*r72*^*/Df(3L)GS1a; simGAL4/UAS-mSox2*^*EnRep*^ .

Since *slit* is a Dichaete target gene in the midline, we examined Slit in DN expressing conditions and found, as expected, a reduction in expression (Figure 1M-P). This observation indicates that, at the level of an individual target gene, the DN constructs mimic *Dichaete* loss of function. However, with both FasII and BP102 staining, the phenotypes we observed were more severe than those exhibited by *Dichaete* null mutants. This suggests that the DN constructs affect processes additional to those regulated by Dichaete and to test this we expressed DN constructs in the midline of *Dichaete* null mutants. Whereas the wild type Sox proteins rescue the CNS phenotypes, expression of DN constructs produced phenotypes more severe than those of *Dichaete* mutants (Figure 2), supporting the view that the DN constructs, in addition to disrupting normal Dichaete function, are likely to have additional effects and thus act as neomorphic alleles.

**Figure 3:**
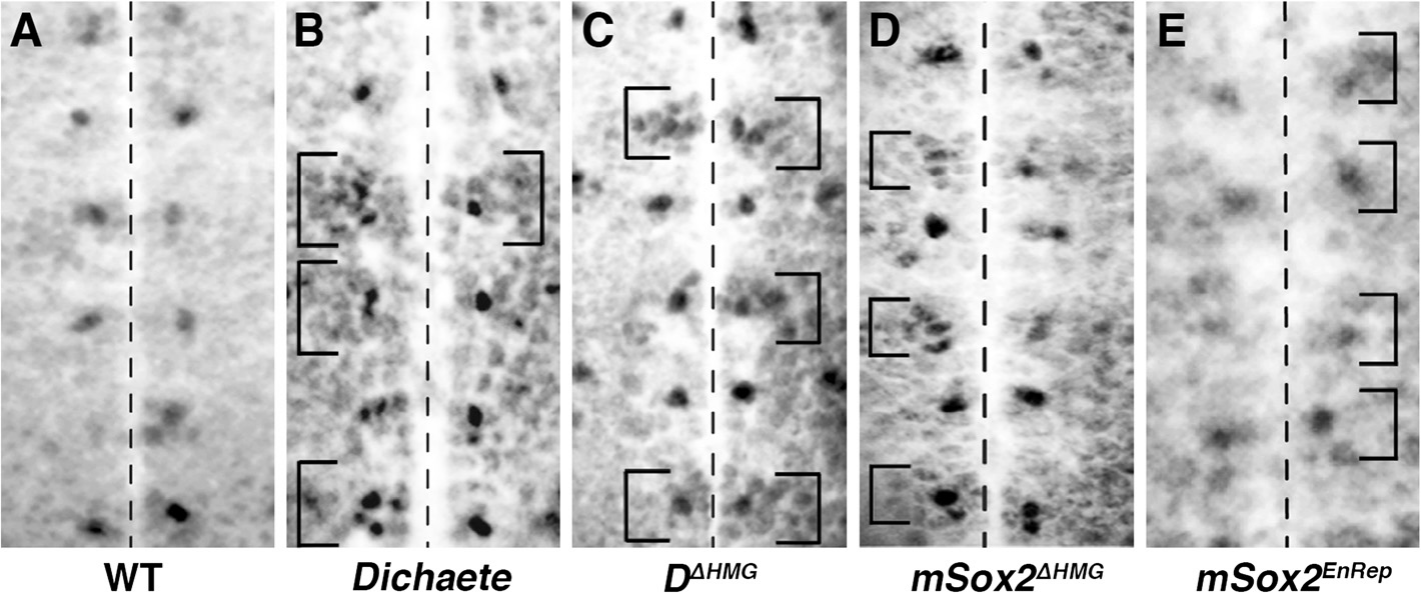
**Proneural gene expression. ** Ventral views of the medial and intermediate columns of the stage 11 embryonic neuroectoderm stained with anti-Achaete, anterior is to the top. The ventral midline is indicated by the dashed line and neuromeres in individual hemisegments indicated by brackets. **A**) Wild type; **B**) *D*^*r72*^*/Df(3L)fz-GS1a*; **C**) *pros-GAL4*; *UAS-D*^*ΔHMG*^; **D**) *pros-GAL4*; *UAS-Sox2*^*ΔHMG*^; **E**) *pros-GAL4*; *UAS-Sox2*^*EnRep*^ .

Since previous studies indicate that Dichaete acts to repress *achaete* expression in the neuroectoderm, we used the DN constructs to investigate the effects on *achaete* expression in the early neuroectoderm using a *prosGAL4* driver [31]. In the stage 11 embryo, *ac* is expressed in a single medial column neuroblast in each hemisegment [32]. Consistent with previous reports [16,17], in *Dichaete* mutants we see a variable increase in the number of *ac* positive cells in the medial column and the appearance of cells in the intermediate column, with up to 5 cells observed in a single hemisegment as well as a more general diffuse staining indicative of low level derepression in the neuroectoderm (Figure 3A and B). Driving the Dichaete or mSox2 ΔHMG constructs with *prosGAL4* results in a similar increase in *ac* positive cells, with both fly and mouse proteins behaving similarly (Figure 3C and D). In contrast, when constructs carrying the EnRep fusions are expressed under the same conditions we see a loss of *ac* expression in the medial column and no ectopic expression in the intermediate column (Figure 3E; Table 1). In addition, we found that expressing ΔHMG in the early embryo resulted in precocious expression of *ac*, with 50-60% of Dichaete^ΔHMG^ or Sox2 ^ΔHMG^ expressing embryos (n = 50) showing weak *ac* expression at stage 6/7 in contrast to wild type expression at stage 8. These observations support the view that Dichaete directly regulates *ac* and further confirm that the DN constructs behave as expected. Specifically, we note that expression of the EnRep construct results in a loss of *ac* expression consistent with its expected activity repressing targets genes. In contrast, the ΔHMG construct results in ectopic expression of *ac* in both medial and intermediate columns, consistent with the idea that it interferes with wild type Dichaete function and supports the idea that Dichaete normally acts as a repressor of gene expression in some circumstances.

**Table 1:** **Neurectodermal expression of*ac*under different*Dichaete*mutant conditions at two different stages of CNS development**

	**Stage 9-10**	**Stage 11**
*Dichaete*^*-*^	Intermediate (8%)	Medial up/extra (42%)
*pros-*GAL4; UAS-*Dichaete*^*ΔHMG*^	Intermediate (35%)	Medial up/extra (25%)
*pros-*GAL4; UAS-*Dichaete*^*EnRep*^	Medial down (50%)	Medial down (ND)
*pros-*GAL4; UAS-*mSox2*^*ΔHMG*^	Intermediate (20%)	Medial up/extra (42%)
*pros-*GAL4; UAS-*mSox2*^*EnRep*^	Medial down (45%)	Medial down (ND)

**Intermediate** = fraction of hemisegments expressing *ac* in the intermediate column. **Medial down** = fraction of hemisegments showing loss of *ac* expression in the medial column. **Medial up/extra =** fraction of hemisegments showing stronger *ac* expression or extra *ac* expressing cells in the medial column. In all cases n > 40 embryos. ND = not done.

### Dichaete regulates *comm* in the midline

To date only two direct Dichaete target genes, *slit* and *ac*, have been identified and to learn more about how Dichaete regulates gene expression in the developing CNS we sought to identify additional direct Dichaete targets. To do this we examined gene expression data, DNA binding data and the literature to identify candidates genes for further study, focusing on genes known to be expressed in the midline or early neuroectoderm.

**Figure 4:**
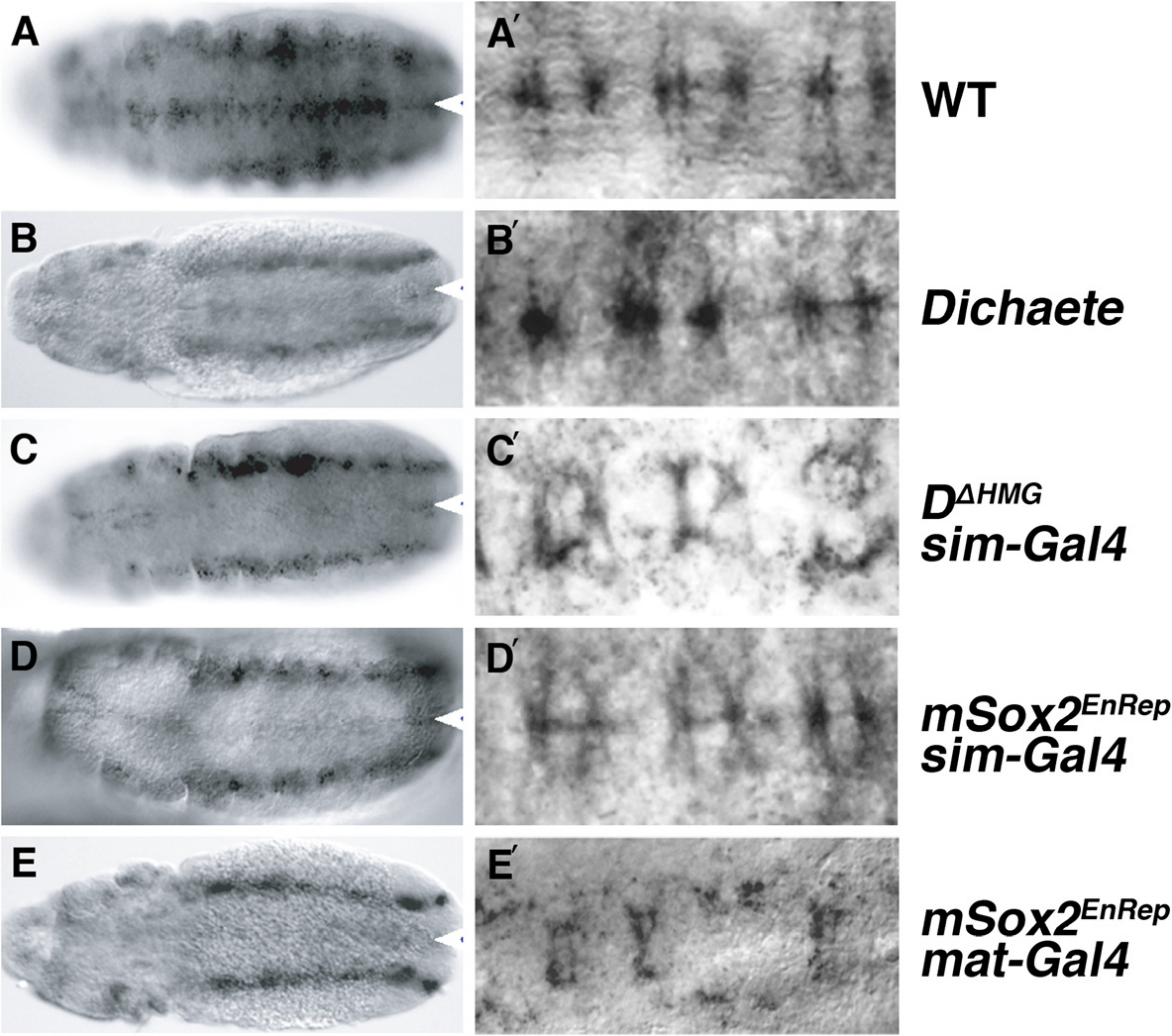
***Commisureless *is a Dichaete target in the midline. ** Ventral views of stage 12 (**A-E**) and close-up of the midline in stage 14 (**A’ – E’**) embryos stained with anti-Comm. Anterior is to the left and the white arrowheads indicate the midline. **A and A’**) Wild type; **B and B’**) *D*^*r72*^*/Df(3L)fz-GS1a*; **C and C’**) *sim-GAL4; UAS-D*^*ΔHMG*^; **D and D’**) *sim-GAL4; UAS-mSox2*^*EnRep*^; **E and E’**) Maternal*-GAL4*^*VP16*^*; UAS-mSox2*^*EnRep*^ .

Dichaete acts directly in the midline, and in particular is known to function in midline glia [15,19]. The *commisureless* gene is expressed in midline glia [33] and in *Dichaete* mutants we observe a loss of Comm expression in the midline of stage 12 embryos (Figure 4A and B). Significantly, lateral epidermal stripes of Comm expression as well as earlier blastoderm and neuroectodermal expression (not shown) are not affected in the *Dichaete* mutants, suggesting Dichaete does not generally regulate *comm* expression in the embryo.

The hypothesis that *comm* is a direct target of Dichaete in the CNS was supported by experiments using the DN constructs: expressing ΔHMG or EnRep proteins in the midline results in the loss of Comm expression along the length of the midline (50-70% of embryos, n > 200/genotype; Figure 4C and D). Further to this, strong and ubiquitous expression of mSox2^EnRep^ via a maternal Gal4-VP16 driver results in a loss of Comm expression in the midline but has little effect on the lateral and early expression of Comm (Figure 4E). This suggests that *Dichaete* interacts with a specific regulatory element of the *comm* gene that control midline expression and does not act as a general activator of *comm* expression. Our observations also further emphasise the functional conservation between Dichaete and Sox2.

Whereas *comm* expression is lost from the early midline in *Dichaete* mutant and DN expressing conditions, by stage 14, when high levels of Comm are detected in midline glia and commissural axons of wild type embryos (Figure 4A’), the effects of loss of Dichaete function are less pronounced. While 75-80% (n > 200) of the Dichaete-ΔHMG embryos show reduced Comm expression, primarily in midline cells and to a much lesser extent in commissural axons, effects are far less pronounced in *Dichaete* null mutants or when EnRep constructs are expressed with *sim-Gal4* (~20% of embryos affected, n > 200; Figure 4B’-E’). Providing EnRep constructs early in development via the maternal Gal4 driver results in a loss of Comm in the midline, and again the effects are much less pronounced in axons. Since axonal Comm expression arises from lateral commissural neurons extending processes to the midline, this expression is likely to be regulated by enhancers distinct from those controlling midline expression. We expect that the reduction in axonal Comm expression we observed at the midline is therefore most likely due to a general disruption of the midline by, for example, the loss of Slit. Together our observations suggest that most *Dichaete* mutant embryos are able to establish glial expression after stage 12 in the absence of Dichaete and suggests that a different, Dichaete independent, regulatory element may control the early and late aspects of *comm* expression. Taken together with the observation on lateral and early *comm* expression, our experiments emphasise the modularity of regulatory sequence organisation and suggest that there is little crosstalk between the enhancers controlling specific facets of *comm* expression.

### Dichaete-mediated regulation of *ase* in neuroblasts

The expression of the Achaete-Scute Complex (AS-C) genes *ac, l’sc* and *sc* in *Dichaete* and *SoxN* mutants has already been described [9,10,16,17]. However, the effects on the fourth AS-C gene *asense* (*ase*) have only been described in *SoxN* mutants [9], despite also being a potential Dichaete target gene in the developing neuroectoderm. We therefore examined the effects of *Dichaete* mutations and DN constructs on its expression*.*

**Figure 5:**
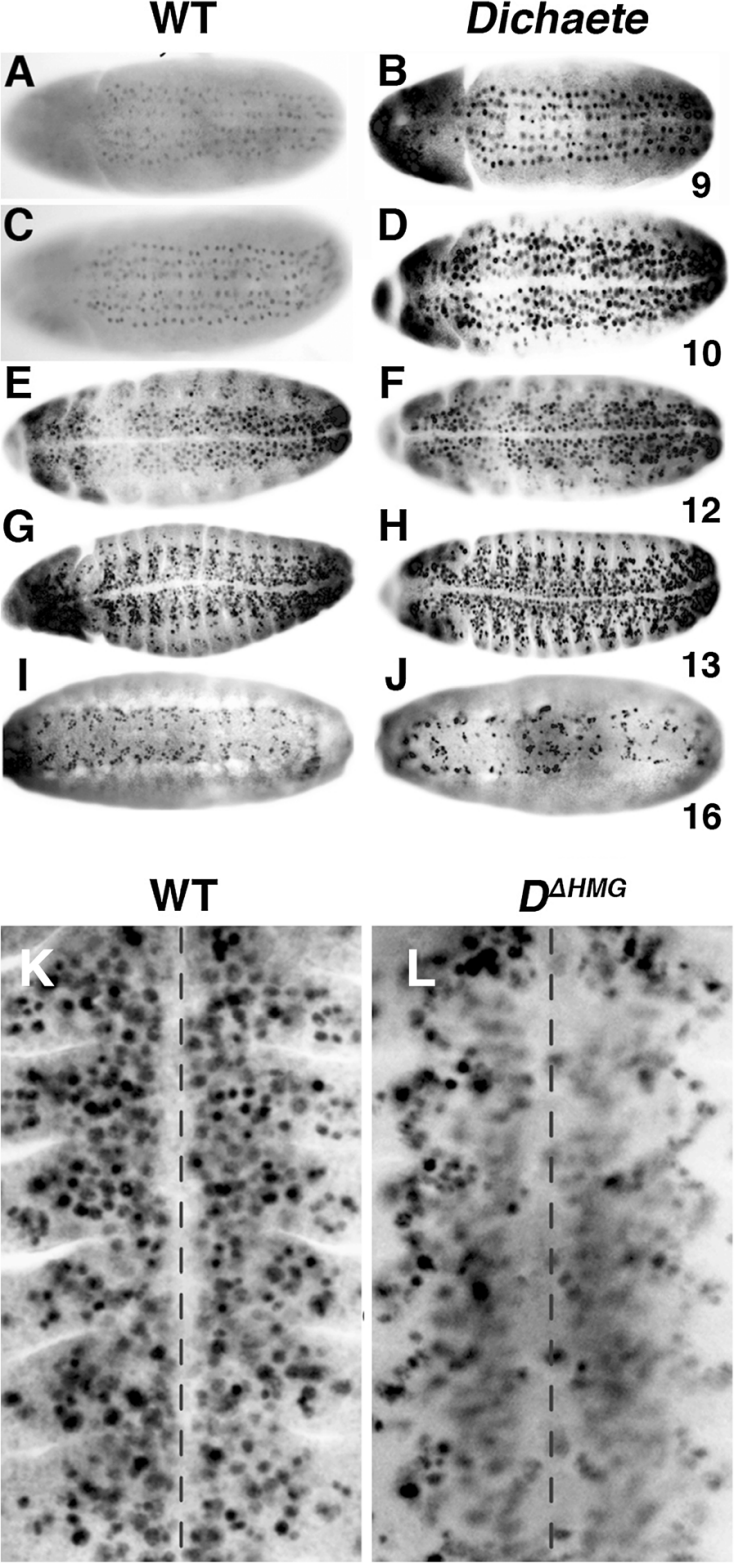
**Effects of Dichaete on Asense expression. A – J**) Ventral views of anti-Ase staining at the indicated stages from 9 to 16 in wild type (**A, C, E, G** and **I**) and *D*^*r72*^*/Df(3L)fz-GS1a* (**B, D, F, H** and **J**). Anterior is to the left. **K and L)** Ventral views of stage 12 embryos stained with anti-Ase. Anterior is to the top and the midline is indicated by the dashed line. **K**) Wild type, **L**) *pros-GAL4*; *UAS-D*^*ΔHMG*^ .

In the wild type embryo, *ase* is initially expressed in CNS neuroblasts in three well-defined columns at late stage 9 [34]. In *Dichaete* null mutant embryos (Figure 5A-J), the expression of Ase is detected earlier and is stronger at stages 9 and 10 than in wild type. After stage 10, the intensity of Ase expression shows no apparent difference between wild type and *Dichaete* mutants. At later stages, the Ase expression pattern becomes disrupted, reflecting, at least in part, the segmentation and CNS disruptions characteristic of *Dichaete* mutants. Thus, as with *ac* in the intermediate column, Dichaete appears to act as a repressor of *ase*. However, in contrast to the results with *ac*, the effects on *ase* expression differ between *Dichaete* mutants and the DN-Sox constructs. With Dichaete^ΔHMG^ driven by *pros-Gal4*, we observe a general loss of the Ase expressing cells in neuroectoderm in 10-15% of embryos, particularly obvious in the medial and intermediate columns after stage 11 (Figure 5K and L). Embryos expressing the other Sox DN constructs show much less severe phenotypes.

### Dichaete binding at target genes

**Figure 6:**
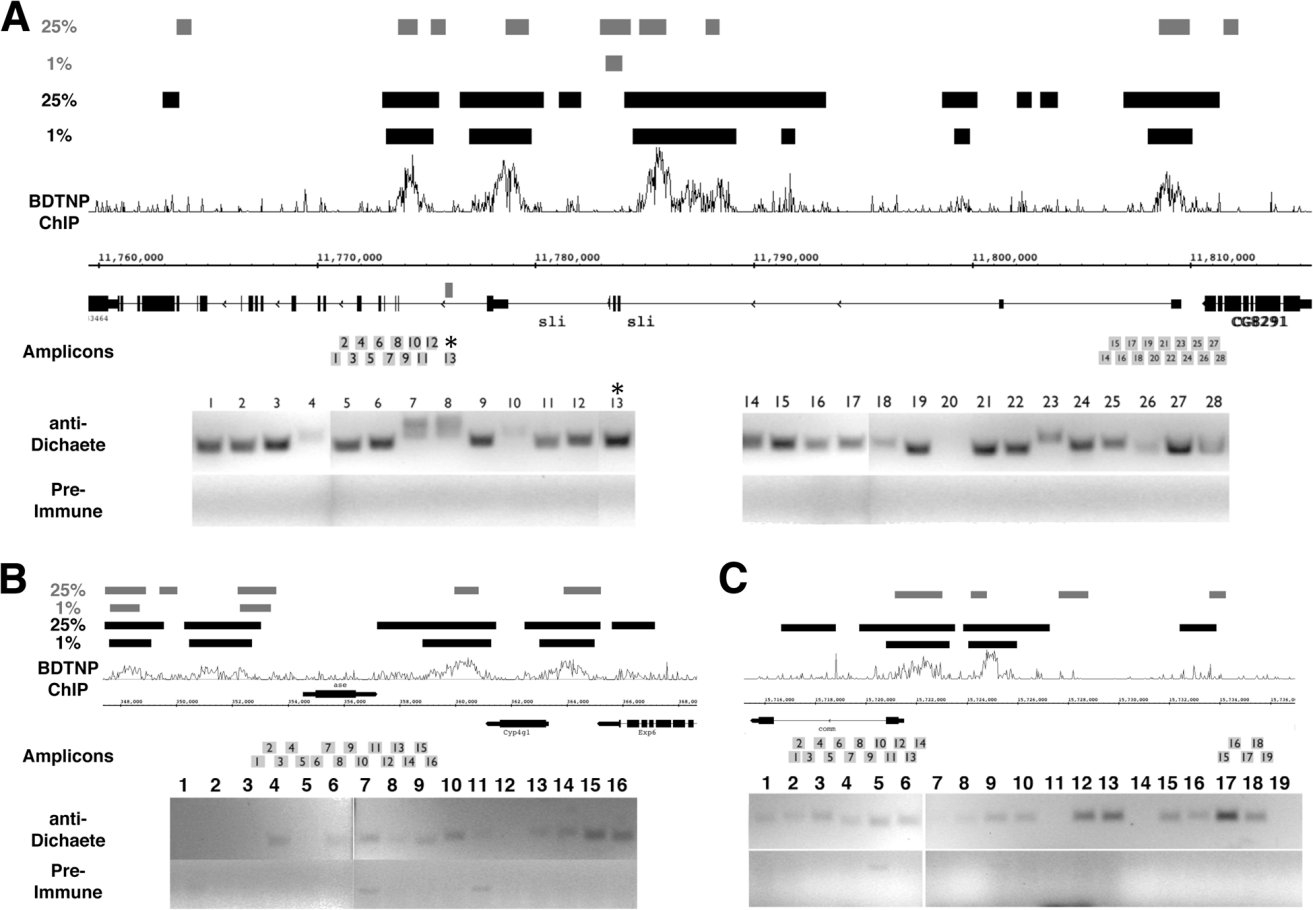
**ChIP assays detect Dichaete binding. ** Binding profiles of Dichaete in the early embryo (stage 5) generated by the Berkeley Drosophila Transcription Network Project [23] are shown in each graph with bound regions identified at 1% and 25% FDR shown as black bars above the ChIP profile. The grey bars represent 1% and 25% FDR regions identified by modENCODE (embryonic stages 1–12). The location of each amplicon assayed by specific ChIP-PCR assays is indicated by the numbered grey boxes below the GBrowse gene model for each region. Gene models above the chromosome scale line are transcribed from left to right and those below from right to left as indicated by the arrows in the introns. The PCR enrichments from anti-Dichaete and control immunopurification reactions are shown in the gel images below the gene models with the numbers indicating the amplicons. **A**) Approximately 55 kb encompassing the *sli* gene. The location of the known *sli* midline enhancer (fragment 13) is indicated by an asterisk and a grey bar above the gene model. **B**) 25 kb around the *ase* gene. **C**) 25 kb around *comm*.

To strengthen the view that Dichaete directly regulates *comm* and *ase*, we examined the genome-wide ChIP-array binding profiles generated by BDTNP [23] and the modENCODE project [24] and found binding intervals identified at 1% false discovery rate (FDR) cut-offs. The BDTNP data were generated using chromatin from 2–3 hr (stage 5) embryos and the modENCODE data from 0–8 hr (stage 1–12) embryos. Together these data may not entirely reflect aspects of late Dichaete binding. We therefore performed specific ChIP-PCR assays around the *ase* and *comm* genes as well as the known target *sli* using chromatin extracted from 0–12 hr embryos (up to stage 14). In the case of *sli*, the BDTNP data shows 6 high confidence binding intervals distributed across various introns and we assayed two of these regions with a series of ChIP-PCR assays (Figure 6A). Using a series of 28 primer pairs encompassing a region around the known midline enhancer [19] and a region spanning the 5’ end of the gene, we find evidence for extensive Dichaete binding that is as broad, if not broader, than the binding regions identified using ChIP-array. Encouragingly, the strongest enrichment was observed with primers covering the known midline enhancer (fragment 13). In the case of *ase*, the BDTNP data identified four regions, two 5’ and two 3’ to the transcription unit. We found the strongest ChIP-PCR signals from primers encompassing the immediate 3’ region (fragments 13–16; Figure 6B) but also found weak signals across the body of the genes. In the case of *comm*, we found the strongest ChIP-PCR signals at the immediate 5’ end of the gene (fragments 12 & 13) and also found evidence for Dichaete binding further 5’, partially overlapping a region identified in the BDTNP and modENCODE data (fragments 15–18; Figure 6C). While there is agreement between the ChIP-array and PCR-based assays, there are clearly some differences. The BDTNP data from the blastoderm stage appears to identify much broader binding than modENCODE, and we note that we see evidence for considerable low level amplification in the PCR assays, with many fragments producing weak but detectable products in comparison to the pre-immune control. These differences may simply reflect the different embryonic stages used in each study or reflect differences in crosslinking conditions. While higher resolution ChIP-seq data will be required to adequately define the extent of Dichaete binding at particular regulatory elements, our experiments confirm that Dichaete is directly associated with the potential targets genes.

Taken together with the evidence from immunohistochemistry experiments on the potential targets in *Dichaete* mutants and DN conditions, these data strongly suggest that *ase* and *comm* are direct Dichaete targets in the embryonic CNS. Interestingly, the *Ascl1* and *Slit1* genes in mouse, which are orthologous to *ase* and *sli* respectively, are associated with Sox2 binding [35], hinting that, along with the functional conservation displayed by Dichaete and Sox2 [15], some of their genetic networks in neural precursors may also be conserved.

### Tissue-specific expression profiling

While candidate gene approaches are useful for identifying a small number of target genes, genomics methods can offer a more global perspective. To this end, we used the dominant negative constructs to perform genome-wide gene expression studies to identify a broader range of potential Dichaete target genes in specific subsets of the CNS. Microarray gene expression analysis was performed with RNA extracted from whole stage 9 embryos expressing the Dichaete DN constructs in the ventral midline or in neuroblasts; the wild-type construct was also included to identify any effects of Dichaete overexpression in the tissues in question. Each construct was expressed in the midline with *sim*-Gal4 and in neuroblasts with *pros*-Gal4, and four biological replicates were performed for each experiment. A UAS-*gfp* construct was crossed to both Gal4 drivers in parallel with the *Dichaete* lines to provide control embryos. Using our standard microarray analysis, a number of differentially expressed genes were detected (Table 2).

**Table 2:** Number of upregulated and downregulated genes detected by microarray analysis using the indicated genotypes

**Genotype**	**Upregulated**	**Downregulated**	**Total**
*sim-*GAL4; UAS-*Dichaete*^*ΔHMG*^	50	119	169
*sim*-GAL4; UAS-*Dichaete*^*EnRep*^	13	35	48
*sim*-GAL4; UAS-*Dichaete*^*WT*^	17	28	45
*pros-*GAL4; UAS-*Dichaete*^*ΔHMG*^	66	533	599
*pros-*GAL4; UAS-*Dichaete*^*EnRep*^	119	154	273
pros-GAL4; UAS-*Dichaete*^*WT*^	18	66	84

The thresholds used to identify differentially expressed genes were average M-value of < −0.5 or > 0.5, p-value < 0.05.

**Figure 7:**
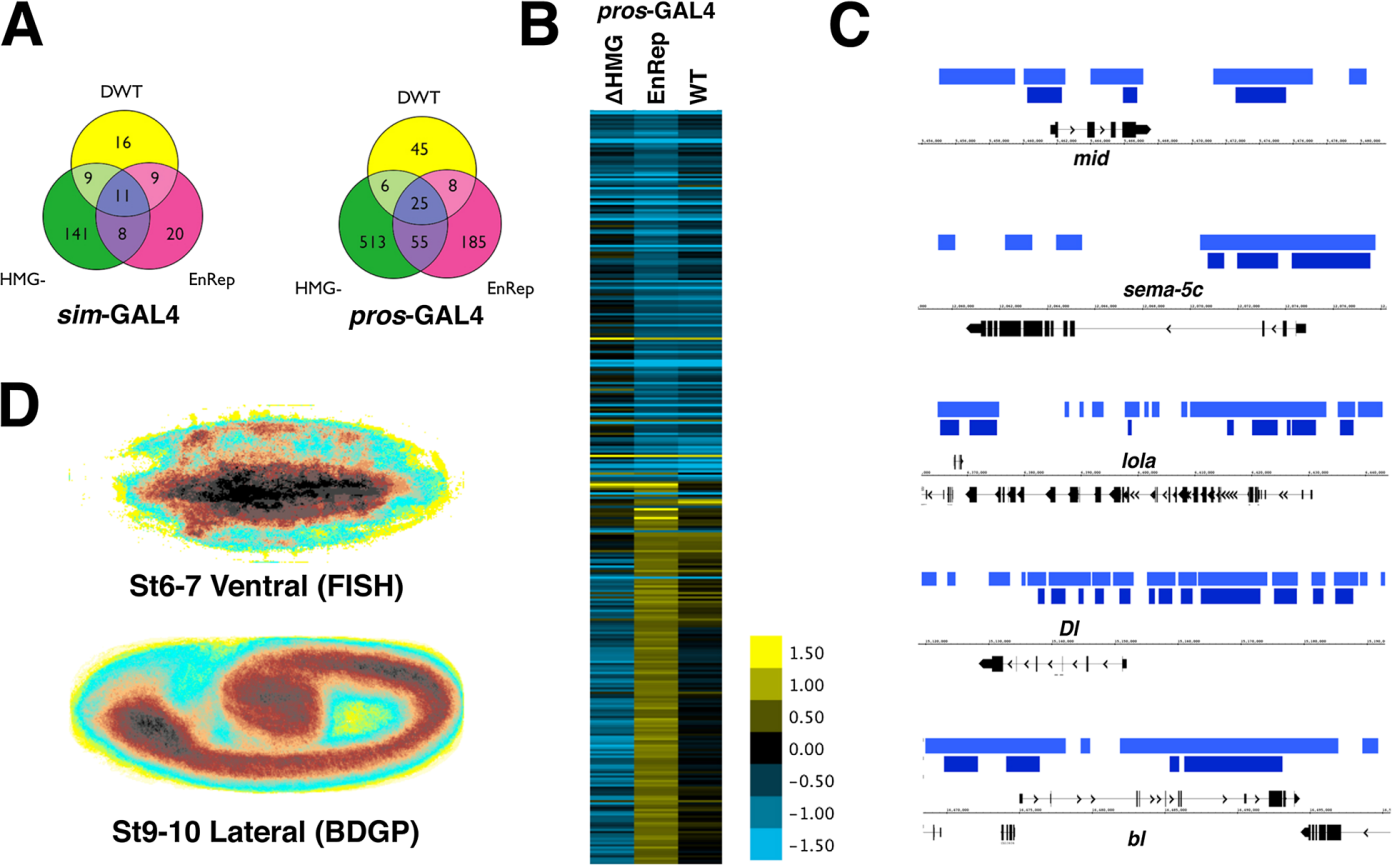
**Gene expression profiling DN mutants. A**) Overlap between gene lists in the *sim*-GAL4 and *pros*-GAL4 experiments. **B)** Hierarchical clustering of genes with significant expression changes when DN proteins are driven in the neuroectoderm with *pros*-GAL4. **C)** Dichaete binding associated with genes changing expression in the *pros-*GAL4 study: dark (1% FDR) and light blue (25% FDR) bars represent binding intervals identified in the BDTNP Dichaete ChIP study [23]. **D**) Gene Expression Maps from FlyExpress [37] .

Across the six microarray studies, we found a total of 994 genes with a significant change in expression in at least one of the experiments. Focusing first on the ventral midline, 214 genes in total changed expression in at least one of the experiments driving a *Dichaete* construct with *sim-*GAL4 (Additional file 1), with the ΔHMG construct showing a stronger effect (169 genes) than the EnRep (48 genes) or wild type (45 genes) constructs. A total of 11 genes are common to all three experiments, and 8 are common to both DN expressing conditions (Figure 7A), including *bancal*, *photorepair* and *Cyp4p2*, which are downregulated. The gene *bancal* was flagged as a target in all 6 gene expression studies, the BDTNP ChIP data shows Dichaete binding around it, and it is annotated with gene ontology (GO) terms “transcription factor binding” and “mRNA binding”, as well as “cell proliferation” and “cell fate commitment”, making it an interesting potential target of Dichaete. *Myosin61F*, *Ras-related protein* (*Rala*) and *Cap-H2* are upregulated, suggesting they may be indirect targets. While we did not identify a significant change in *sli* expression, we did find 6 genes identified in a screen for midline expressed genes, including *kek1*, *Kr-h1*, *Sema-5c* and *nemy*[36]. The comparatively small number of gene expression changes we identified is likely to be a reflection of the fact that the ventral midline is a very small component of the embryo and thus only very pronounced changes in gene expression are expected to be detected by the microarray analysis.

In the neuroblast experiment, driving the DN constructs with *pros-*GAL4, we identified a total of 851 genes that changed expression in at least one of the experiments (Figure 7A; Additional file 2). It was not unexpected that this experiment affects a larger number of genes since the tissue targeted is larger than the midline and consequently it is easier to pick up more subtle changes in gene expression. Again the ΔHMG construct showed the strongest effect (599 genes) with a smaller number of changes detected when expressing for the EnRep (273 genes) or wild type (84 genes). While we might have expected the EnRep construct to consistently downregulate genes, we were surprised to find that 30-40% of misregulated genes showed significant upregulation (Figure 7B; Table 2). Focusing on the genes changing expression in both the DN conditions, we identified 80 genes and of these 74 were downregulated in the presence of the EnRep construct. We found that 15 of these have GO annotations associated with proteolysis and while we considered that this could be a stress or cellular damage response, we note that 14 of these genes were downregulated by the expression of the ΔHMG construct, suggesting they require Dichaete function for their activation.

We identified 69 genes that changed expression in both the midline and the neuroblast experiments, suggesting they are good candidate target genes. While we did not identify any significantly enriched gene ontology terms associated with this set of genes, we did find 30 terms linked to development with corrected p-values lower than 0.01 when we examined the overall list of list of 994 differentially regulated genes (Additional file 3). To find further evidence supporting direct Dichaete regulation, we compared the set of 994 genes with published genome-wide ChIP datasets [23,24] and found that 503 (51%) of these have evidence for Dichaete binding, suggesting that a substantial fraction of the genes we identify may be genuine Dichaete targets (Figure 7C, Additional file 4). Significantly, when we examined the list of 503 genes showing expression changes and evidence of Dichaete binding in the ChIP study, 60 development related ontology terms were found with much lower p values (1.00E-09), including ontology terms associated with nervous system development (GO:0007399) and generation of neurons (GO:0048699).

Of these 503 Dichaete bound genes, 107 have *in situ* based gene expression data available in the FlyExpress database [37]. Creating a Gene Expression Map from these genes shows an enrichment in the ventral neuroectoderm and midline early in embryogenesis, and very strong CNS enrichment at Stage 9–10, when the gene expression profiling was performed (Figure 7D). This suggests that the genes showing expression changes and Dichaete binding are likely to constitute bona fide Dichaete targets. We submitted the list of Dichaete bound genes to i-*cis*Target [38] to search for enriched transcription factor binding site matrices and identified a Dichaete-like AACAA motif (a consensus Dichaete binding motif is AACAAT [39]) associated with 45% of the genes (11th of 127 enriched motifs, e-score = 4.12). Interestingly, we also found enrichment for motifs associated with TFs identified as potential Dichaete targets (Lola, e = 3.22; Hb, e = 2.82 and E(bx), e = 2.76), hinting at potential regulatory interactions.

These new Dichaete target genes include a number of transcriptional regulators (e.g. *achaete*, *bunched*, *d4*, *E2f*, *hunchback*, *huckebein*, *longitudinals lacking*, *midline*, *mushroom body defective*, *p53* and *SoxN*) as well over 100 genes annotated with the GO term signalling, including several components of the Notch (*e.g. Delta, Hairless, Notchless, bunched*), Egfr (*Cbl, csw, sno, sty*), Wnt (*fz, fz3, nkd, pygo*) and JAK-STAT (*hop, Stat92E*) pathways. We noticed that 20 of the new target genes are annotated as being involved in lateral inhibition, further implicating Dichaete in the early events in the neuroectoderm. If Notch signalling is compromised in the early neuroectoderm, lateral inhibition breaks down and excess neural cells are produced at the expense of epidermis [40]. In our analysis of *ac* expression we found extra Ac-positive cells in the medial column consistent with a reduction in lateral inhibition (Figure 3). In particular, we noticed that while expression of EnRep constructs generally results in loss of *ac* expression, we did observe hemisegments with increased numbers of *ac*-positive cells (Figure 3E). As well as these early events in neural development, we found 50 genes involved in neuron differentiation (*i.e.* axonogenesis, axon fasciculation and guidance, dendrite morphogenesis), suggesting that Dichaete acts at the level of terminal differentiation genes.

Focusing on aspects of neural development, we found that 18% (89 genes) of the bound and mis-regulated genes have CNS development GO annotations (corrected p-value = 0.001). Analysis of this set of 89 genes with the STRING interaction database [41] reveals a highly connected network centred on the Wnt and Egfr pathways, further emphasising the Dichaete role in regulating multiple signalling pathways during nervous system development (Additional file 5). Taken together, our expression analysis combined with published Dichaete binding data identify a large number of likely direct targets. Clearly these genes will need further validation, however the lists provide a platform for the further analysis of group B Sox activity in subsets of the CNS. Our experiments also emphasise that ideally data from multiple experiments is needed when interpreting results generated by experiments expressing dominant negative proteins due to the lack of sensitivity and existence of off-target effects.

## Discussion

In this study we have investigated the use of dominant negative alleles of the Sox domain transcription factor Dichaete to identify tissue-specific functions and potential target genes. We show that expression of Dichaete alleles with a deleted DNA binding domain or fused to a strong transcriptional repressor in subsets of the embryonic CNS produce Dichaete-like phenotypes. Analysis of predicted target genes in the CNS midline (*comm*) or neuroectoderm (*ase*) suggest that Dichaete directly regulates these genes as judged by molecular phenotypes observed both in *Dichaete* mutants and when exposed to the DN constructs. Expression profiling embryos expressing DN constructs in the CNS midline or in neuroblasts identifies approximately 1000 genes showing altered expression. While some of the identified gene expression changes will be attributable to off-target effects of the DN constructs, we were encouraged to find that half of misexpressed genes are associated with *in vivo* Dichaete binding, indicating that we have identified a substantial repertoire of new Dichaete targets.

We validated the activity of the DN constructs on the established Dichaete midline target gene *slit*, showing that expression of both proteins in the midline led to loss of *slit* expression and *Dichaete*-like midline phenotypes. Similarly, in the neuroectoderm we confirmed previous work showing that *ac* is a direct Dichaete target [16,17] with the ΔHMG construct leading to derepression of *ac* in the medial column of the neuroectoderm, expression of the EnRep fusion leading to *ac* repression and ChIP-PCR assays with Dichaete antiserum enriching regulatory sequences in the *slit* gene.

We went on to identify two new Dichaete targets in the CNS, *comm* in the midline and *ase* in the neuroectoderm. In midline glia, a cell type where Dichaete is known to control *slit* expression, the *comm* gene is also expressed in the midline although its function here is not understood [42]. Targeting DN expression to the midline, we found a loss of Comm expression. Significantly, we also found that when EnRep fusions were expressed throughout the embryo via a maternal GAL4 driver, only midline Comm expression was affected while lateral domains were unaffected. These observations indicate that the action of DN Dichaete constructs on *comm* is not a general effect, but instead targets specific regulatory elements. Again we found support for direct Dichaete action by analysing published Dichaete ChIP-array data and performing specific ChIP-PCR assays.

These observations suggest that Dichaete is only involved in the initial establishment of *comm* expression in the midline, since later in development *comm* becomes Dichaete independent. This is analogous to our previous finding that Dichaete is required for the establishment of primary pair-rule gene expression at the blastoderm stage, but that after cellularisation these genes are expressed independently of Dichaete [5]. This difference between expression establishment and maintenance has previously been described for the Runt repressor and may be a more widespread feature of transcription factor biology [43]. However, arguing against Sox-independent late *comm* expression, we note that stage 12, when *comm* expression recovers in *Dichaete* mutants, is when the *SoxN* gene first becomes active in the midline [9], suggesting loss of Dichaete may rescued by SoxN expression. Since we find that late *comm* expression is affected in ΔHMG expressing embryos and when the EnRep fusions are supplied ubiquitously, it is possible that Dichaete and SoxN use the same co-factors to regulate *comm.*

In the neuroectoderm we found that *ase*, a member of the AS-C that acts as a neural precursor gene [34,44], is derepressed in *Dichaete* mutants. We found earlier than normal expression of *ase* in the early neuroectoderm along with evidence for Dichaete binding in ChIP assays. Thus, like the proneural AS-C complex member *ac*, Dichaete appears to act as a repressor. Whether this is also in conjunction with the Ind and Vnd homeodomain proteins remains to be determined. Interestingly, in *Dichaete* mutants we observe derepression of *ase* in all three columns of the neuroectoderm, whereas Dichaete expression is restricted to the medial and intermediate columns after gastrulation. This observation suggests that earlier Dichaete expression at the blastoderm stage may influence prepatterning throughout the neuroectoderm. We expect that expression of the EnRep constructs would have little effect at stage 9–10 since *ase* expression is already very low in the wild type at this stage. It was also observed that expression of ΔHMG constructs has little effect during early stages of development. There are a number of possible reasons for this: most likely Dichaete mediated *ase* repression occurs earlier in development than the activation of the *pros-Gal4* driver, alternatively the ΔHMG constructs might be unable to sequester the relevant co-factors. It is also possible that *ase* may be an indirect target, however our other evidence suggests this is not the case. We found severe disruption of *ase* expression when all of the DN constructs are expressed via a maternal Gal4 driver (not shown), however it is likely that this is due to pleiotropic effects of inhibiting Dichaete function.

Our analysis uncovered a number of potential off-target effects resulting from the expression of both versions of DN Dichaete. In particular we note that phenotypes tended to be stronger than those observed in *Dichaete* null embryos and we also saw stronger phenotypes when expression of DN constructs was performed in a *Dichaete* null background. We do not attribute these effects to misexpression *per se*, since expression of wild type Dichaete under the same conditions resulted in very mild phenotypes and also rescues *Dichaete* null phenotypes. In addition, expression of wild type Dichaete resulted in a small number of significant gene expression changes, presumably reflecting some dosage-dependent effects of Dichaete. Unexpectedly from the phenotypic analysis, we found that both in the midline and in neuroblasts the ΔHMG construct affected the expression of twice as many genes as the EnRep construct. Expression of ΔHMG mostly led to down regulation of transcript levels, with 70-90% of significantly changed genes repressed compared to controls. We imagine that the ΔHMG protein acts by sequestering DNA binding proteins or other cofactors that normally interact with Dichaete but that this will also result in these cofactors being unavailable for interaction with other TFs and thus affect other, Dichaete-independent, processes. For example, we know that Dichaete interacts with Vvl and Sim in the midline [15,19], however, both these proteins play much more extensive roles in embryonic development and, in particular, show stronger midline phenotypes than Dichaete mutants [45,46]. Similarly, Dichaete is known to interact with Ind [16,47] in the neuroectoderm, but only in a subset of this transcription factor’s expression domain. Therefore at least some of the effects we see from expression of the ΔHMG will be attributable to direct action on Dichaete targets.

In the case of the EnRep fusion, we anticipated that binding of this protein to Dichaete targets in the genome would result in the repression of these genes [30], a view reinforced by preliminary data from expression profiling Dichaete null mutant embryos indicating that Dichaete mainly acts as a transcriptional activator (JA and SR, unpublished observations). We were therefore surprised to find as much as 40% of misregulated genes identified in the EnRep screens showed increased expression compared to controls. These observations may indicate a substantial level of off-target effect, where upregulated genes reflect secondary changes in expression due to repression of a gene encoding a repressor. Alternatively, the upregulation may result from unanticipated interactions between the regulatory proteins binding together at cis-regulatory elements such that the effects of the EnRep domain are masked. Finally, it is possible that in the case of some highly expressed genes the Engrailed Repressor domain may not function and the EnRep construct behaves like a Dichaete overexpression construct.

While our focused analysis identified two likely Dichaete targets, the published ChIP data and our preliminary *Dichaete* mutant expression profiling suggest that Dichaete may regulate thousands of targets in the *Drosophila* genome, and the expression profiling experiments presented here support this view. While some of the gene expression changes we identify reflect secondary consequences downstream of direct Dichaete targets, we were reassured to find evidence for Dichaete binding associated with over half of the misregulated genes. Significantly, these bound and regulated genes are enriched for nervous system expression and also show significant over-representation of gene ontology terms associated with development and specifically CNS development. A number of genes encoding transcription factors associated with CNS development were identified in the screen (*achaete*, *bunched*, *d4*, *E2f*, *hunchback*, *huckebein, longitudinals lacking*, *midline*, *mushroom body defective*, *p53* and *SoxN*), indicating that Dichaete may play diverse roles in controlling aspects of the regulatory networks underpinning CNS development. We also identified enriched binding motifs for Hb and Lola associated with these bound and regulated genes, hinting at potential regulatory interactions involving Dichaete. Of particular interest is Hb, which is known to be involved in the temporal sequence of TFs that specify neuroblast fates [48]. Dichaete has been shown to participate in a similar process during larval CNS development [22] and our observations suggest that, as well as being expressed together in neuroblasts, there may be a regulatory loop between Dichaete and Hb in the embryo.

Other potentially interesting Dichaete targets include *nejire* (*nej*), encoding a histone acetyltransferase involved in both segmentation and neuron differentiation, *bancal*, encoding a splicing regulator, and several components of the Notch signalling pathway including the genes encoding the Delta ligand and the transcriptional co-repressor Hairless. As we note above, the presence of extra *ac* expressing cells observed when expressing DN constructs is consistent with a loss of lateral inhibition and suggests that Dichaete regulates multiple steps in the neural specification pathway. Group B Sox proteins have previously been shown to regulate Notch signalling in vertebrates by directly regulating the Hes class bHLH genes [28,49] and we identified the Notch pathway repressor encoded by *Hairless* as well as the Bearded-family member *E(spl)m4* as putative direct Dichaete targets. We also identified several components of the Wnt signalling pathway as target genes. Previously both Dichaete and SoxN have been shown to negatively regulate the Wnt pathway in the embryonic epidermis [50,51] and our studies suggest this extends to other tissues. Interestingly, in the epidermis a key target of SoxN/Dichaete is *shavenbaby*, which is regulated by both Wnt and EGFR signalling. Our screen also identified *sprouty* and *Cbl*, encoding two global inhibitors of the EGFR pathway [52], suggesting a more general Sox-mediated interaction between EGFR and Wnt pathways. Sox factors have been proposed to interact with Wnt signalling pathways during a variety of developmental processes [53] and our data suggests that this may occur at multiple levels.

## Conclusion

Taken together our study acts as a starting point for further analysis of the role Dichaete plays in the gene regulatory networks governing the development of the embryonic CNS in *Drosophila*. We validate new Dichaete target genes in the midline and neuroectoderm as well as identifying a set of over 500 Dichaete bound and regulated genes. Given the functional conservation between Dichaete and vertebrate Sox2, as well as the wider association between group B Sox proteins and CNS development, we suggest these data may be more widely applicable to understanding the role Sox proteins play in critical developmental processes.

## Methods

### *Drosophila* stocks

All fly stocks were maintained on standard yeast-cornmeal media at 25°C. The following stocks were used (all nomenclature is according to FlyBase): *D*^*r72*^*/TM6B*, *Df(3L)fz-GS1a/TM3 Sb*^*1*^*, w; p{*GAL4-*pros*^*C20*^*}*C74-17,*w; p{GAL4-sim3.7}2/CyO; p{GAL4-sim3.7}3, w; p{matα4-GAL-VP16}V2H*, *w; p{matα4-GAL-VP16}V37*.

### Generating dominant negative constructs

To generate pUAST-*Dichaete*^*ΔHMG*^, pUAST-*Dichaete*^*EnRep*^ and pUAST-*Dichaete* constructs, cDNA fragments were amplified from a cDNA clone [5] by standard PCR using *PfuTurbo* DNA polymerase (Stratagene) and cloned into the pBluescript II KS (−) vector. For the *Dichaete*^*ΔHMG*^ construct, an N terminal fragment encompassing amino acids 1 to 135 was amplified with primers containing a 5’ *Eco*RI site and a 3’ *Xho*I site, cut with *Eco*RI and *Xho*I, and cloned into pBluescript II KS(−). The C-terminal *Dichaete* fragment, from amino acids 210 to 282. was amplified with primers containing a 5’ *Xho*I site and a 3’ *Kpn*I site, cut with *Xho*I and *Kpn*I, and cloned into the plasmid containing the N-terminal fragment. For the full-length *Dichaete* construct, the insert was amplified from the full-length cDNA clone (GenBank accession X96419 and cloned into the *Hin*dIII and *Kpn*I sites of pBluescript II KS(−). For the *Dichaete*^*EnRep*^ construct, the Engrailed repressor domain (amino acids 1–298) was amplified with primers containing a 5’ *Eco*RI site and a 3’ *Hin*dIII site (Engrailed cDNA kindly provided by B. Sanson, University of Cambridge), cut with *Eco*RI and *Hin*dIII, and cloned into the pBluescript II KS(−) vector containing the full-length *Dichaete* cDNA. Subsequently, *Dichaete* fragments were amplified from the pBluescript plasmids with *PfuTurbo* polymerase and cloned into the pENTR™/D-TOPO vector (Invitrogen) with a Directional TOPO Cloning Kit (Invitrogen). The TOPO entry clones were used to recombine the *Dichaete* fragments in-frame with a C-terminal GFP cassette in a pUAST Gateway destination vector, pUAST-*att*R1-CmR-*ccd*B-*att*R2-cGFP (generated by the Martinez Arias Laboratory, Department of Genetics, University of Cambridge). The UAS mouse *Sox2*, UAS mouse *Sox2*^*ΔHMG*^ and UAS mouse Sox2^*EnRep*^ constructs were kindly provided by Dr. Karine Rizzoti (NIMR, Mill Hill). The UAS m*Sox2* construct contains a full-length wild type mouse Sox2 cDNA fused with a C terminal EYFP tag. The UAS *Sox2*^*ΔHMG*^ construct contains a C-terminal fragment of the mouse *Sox2* cDNA fused with a C terminal EYFP tag followed by a Myc tag. UAS *Sox2*^*EnRep*^ contains an N-terminal fragment of mouse *Sox2*, including the HMG domain, fused to the Engrailed repressor domain at the C terminus followed by a Myc tag. Transgenic flies were generated by standard germline transformation [54].

### Immunohistochemistry

Embryos were collected from yeasted apple juice agar plates, washed thoroughly with water and dechorionated in 50% commercial bleach for 3 min. Dechorionated embryos were fixed and processed for immunohistochemistry with the Vector ABC Elite Kit™ (Vector laboratories) using minor modifications to standard techniques [55]. Stained embryos were mounted in 70% and examined with a Zeiss Axiophot microscope using bright-field or Nomarski optics. Images recorded with a digital camera using Openlab software and processed with Adobe Photoshop. The following antibodies were used at the stated dilutions: mouse anti-Achaete (1:5, Developmental Studies Hybridoma Bank), rabbit anti-Asense (1:10, gift of A. Brand), Rabbit anti-Commisureless (1:50 gift of G. Tear), Mouse anti-Fasciclin II (1:4, Developmental Studies Hybridoma Bank 1D4), mouse anti-Slit (1:10, Developmental Studies Hybridoma Bank C555.6D).

### ChIP assays

Chromatin immunopurification from *Drosophila* embryo collections using Rabbit anti-Dichaete [15] or control IgG was carried out as previously described [56]. Immunopurified chromatin from anti-Dichaete and control reactions was assayed in parallel with oligonucleotide primer pairs that amplify fragments of the regulatory regions of *ase*, *comm* and *slit* (Primer sequences designed with Primer 3). ChIP-array data from [23] was obtained from Array Express (E-TABM-736) and from the modMINE website (E0-8_D | modENCODE_2571) [57]. Binding intervals were visualized using the Integrated Genome Browser [58]. Comparisons between expression data and binding data were made via custom lists in FlyMine [59].

### Microarray analysis

Approximately 150 embryos per sample were collected between 3.5 and 4.5 hours after egg laying and stored frozen in Trizol. Following RNA extraction, reverse transcription, Klenow amplification and labelling, samples were hybridised to INDAC FL002 (GEO: GPL5016) or FL003 (GEO:GPL14121) Drosophila gene expression arrays using our standard protocols (http://www.flychip.org.uk/). Four biological replicates were performed for each experiment, with 2 dye swaps incorporated into the experimental design to control for bias due to different dye incorporation efficiencies. Arrays were quality checked manually, removing any spots that appeared to be affected by high levels of background or artefacts. Our standard data analysis pipeline was employed (http://www.flychip.org.uk/) using Dapple for spotfinding and quantifying signal intensities [60], and the Variance Stabilisation and Normalization (vsn) package in R [61] followed by CyberT [62] to assess statistical significance. The thresholds used to identify differentially expressed genes were average M-value of < −0.5 or > 0.5 with a p-value < 0.05.

## Competing interests

The authors declare that they have no competing interests.

## Authors’ contributions

SPS generated the dominant negative lines, carried out the phenotypic analysis and performed the ChIP-PCR assays. JA performed the microarray and genomics analysis, and helped write the manuscript. SR designed the experiments, analysed data and wrote the manuscript. All authors read and approved the final manuscript.

## Supplementary Material

Additional file 1 **Table of significant gene expression changes observed driving DN Dichaete constructs in the midline with *sim*-GAL4. ** Transcript = FlyBase designated transcript against which the array probe was designed. Gene = FlyBase gene name. M = average log2 fold change. p = p value. Click here for file

Additional file 2 **Table of significant gene expression changes observed driving DN Dichaete constructs in neuroblasts with *pros*-GAL4. ** Transcript = FlyBase designated transcript against which the array probe was designed. Gene = FlyBase gene name. M = average log2 fold change. p = p value. Click here for file

Additional file 3 **Table of enriched Gene Ontology terms associated with a) the list of all 994 genes changing expression across the microarray studies and b) the subset of 503 genes with associated Dichaete binding.** Click here for file

Additional file 4 Table of genes with DN-Dichaete dependent expression changes and associated Dichaete binding intervals. Click here for file

Additional file 5 **Figure showing the output from the STRING database using the 89 Dichaete target genes with Nervous System Development annotations (GO:0007399).** In this network the thickness of the edges represents the confidence in the interaction, with thicker lines showing stronger associations. All nodes show Dichaete binding and expression changes apart from those highlighted: ** Dichaete binding and expression change below significance cut-off, * Dichaete binding only. Click here for file
