# Supplementary figures and images for: Identifying targets of the Sox domain protein Dichaete in the Drosophila CNS via targeted expression of dominant negative proteins
Source: BMC Dev Biol. 2013 Jan 5;13:1. doi: 10.1186/1471-213X-13-1 (PMC3541953; doi:10.1186/1471-213X-13-1)

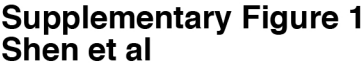

Supplement: Additional file 5 — Figure showing the output from the STRING database using the 89 Dichaete target genes with Nervous System Development annotations (GO:0007399). In this network the thickness of the edges represents the confidence in the interaction, with thicker lines showing stronger associations. All nodes show Dichaete binding and expression changes apart from those highlighted: ** Dichaete binding and expression change below significance cut-off, * Dichaete binding only. [file 1471-213X-13-1-S5.pdf]
